# Can ENGLAND'S National Health System Reforms Overcome the Neoliberal Legacy?

**DOI:** 10.1177/00207314221115945

**Published:** 2022-07-26

**Authors:** Kate Bayliss

**Affiliations:** Department of Economics, SOAS University of London, London, UK

**Keywords:** NHS, financialisation, privatisation, integrated care

## Abstract

England’s National Health Service (NHS) is in the process of major reform as old institutional structures based around an internal “market” are being replaced with integrated care systems. The changes represent a significant shift in ethos away from commercialisation to collaboration between health providers. But the way that these policies unfold will depend on the context within which they are implemented, and three decades of neoliberal reforms have left their mark on the structure of the health system. This paper shows how a powerful, politically-connected financialised private sector has evolved alongside a weakened public system, depleted further by the pandemic. While the share of overall public health spending reaching the private sector has not increased greatly over the past decade, private financial investors are strongly embedded in some segments of health delivery, particularly mental health services where shareholder returns are boosted by financial engineering. The boundaries between private and public are increasingly blurred with the NHS treating private patients and self-payment for health services is increasingly normalised. Rather than traditional privatisation, the health system is facing a more subtle and pernicious erosion of public services across different dimensions which seems likely to continue despite the new reforms.

England's National Health Service (NHS) is in the process of major reform as old institutional structures based around an internal “market” are being replaced with integrated care systems. The changes represent a significant shift in ethos away from commercialization to collaboration between health providers. But the way that these policies unfold will depend on the context within which they are implemented, and three decades of neoliberal reforms have left their mark on the structure of the health system. This article shows how a powerful, politically connected, financialized private sector has evolved alongside a weakened public system, depleted further by the pandemic. While the share of overall public health spending reaching the private sector has not increased greatly over the past decade, private financial investors are strongly embedded in some segments of health delivery, particularly mental health services, where shareholder returns are boosted by financial engineering. The boundaries between private and public are increasingly blurred, with the NHS treating private patients, and self-payment for health services is increasingly normalized. Rather than traditional privatization, the health system is facing a more subtle and pernicious erosion of public services across different dimensions that seems likely to continue despite the new reforms.

England's National Health System (NHS) is in the process of radical restructuring, not only in terms of institutional structure, but in underlying ethos. The 2021 Health and Care Bill (HCB) going through Parliament at the time of writing, in April 2022, emphasizes collaboration and cooperation among health care providers, in sharp contrast to the neoliberal focus of past health reforms. The new structure puts greater emphasis on integrated, place-based population health.^1^ But the bill has attracted considerable criticism for failing to address fundamental staffing constraints and for placing unprecedented powers in the hands of the Secretary of State for Health.^2^ Some campaign groups, such as We Own It and Keep Our NHS Public, and academics^3^ are concerned that the HCB will lead to greater privatization, while other commentators disagree.^4, 5^ To assuage such fears, the bill was amended to prevent private companies from taking a place on integrated care boards.^6^

This article explores these issues from a political economy perspective. It argues that, while there is a dramatic change in policy direction, this follows 30 years of pro-market cultures, rhetoric, and policy, which remain deeply ingrained in many aspects of the health service. Privatization in terms of the transfer of NHS-funded services to the private sector is, so far, limited in scope. However, the increased entry of profit-oriented U.S. health companies and private equity investors has led to the “financialization” of some elements of NHS provision, which are now subject to more aggressive means of profit extraction. In addition, the NHS has itself become a provider of private health services. Moreover, austerity measures from 2010 onward have severely weakened NHS capacity, which has been depleted further by COVID-19. Growing service gaps have led to a surge in self-payment for health services, strengthening the position of private health care in England. Thus, the NHS is at the convergence of a complex set of pressures that are not captured in simple metrics on privatization.

The following section sets out background details of the core elements of the policy change and the conceptual approach of the article. This is followed by a detailed analysis of the nature of financialization in the NHS and the shifting agency relationships across private and state agents. The subsequent section looks at the wider policy context, as well as the effects of austerity and the COVID-19 pandemic on the NHS and relations between the state and private sector. The article then draws these strands together to consider the implications for the HCB before the final section concludes. Overall, the article argues that health reforms need to be understood in terms of shifting agency relationships set within historically evolved structures and processes. While the HCB appears to present a shift away from market-oriented health systems, much of the underlying neoliberal culture remains, and an increasingly powerful private sector is now enmeshed in the provision of health services in complex ways.

## Background and Approach

England's NHS was established as a publicly funded and provided universal system in 1948. But since the 1990s, successive reforms and restructurings have incorporated private-sector financial and commercial logics into the public health system.^7^ The institutional framework has been oriented around an internal “market,” with Clinical Commissioning Groups made up of local general practitioner (GP) practices and other clinicians acting as the “buyers,” and NHS trusts covering hospital, mental, community, and ambulance health services as the “sellers.” Since 2003, sellers have also included independent-sector providers. Patients are allowed to choose their hospital for some procedures, including for-profit providers.^8^ Under the 2012 Health and Social Care Act (HSCA), Clinical Commissioning Groups were in some cases required to allocate funds by means of competitive tenders, with both private and NHS providers entitled to bid.^9^ Funds were allocated via a complex “tariff” system known as “payment by results,” on the basis of equivalent units of health care administered. This health care model, based on competition and a pseudo market, attracted growing criticism. It was costly to run, the rules were confusing, and the NHS, operating on limited resources, was at a disadvantage in taking part in tendering exercises. The NHS was vulnerable when a contract was lost to the private sector as this risked losing a vital revenue source.^10^

Pilot measures to introduce a new organizational framework led to the 2019 NHS Long-Term Plan,^11^ which was the basis for the 2021 HCB. The new approach is oriented around collaboration and cooperation between providers, rather than units of health care.^12^ Under the proposed legislation, health care services in England will be organized through 42 regional integrated care systems, governed by two new bodies: integrated care boards (statutory NHS bodies made up of area-based NHS agencies and other health providers that will govern most of the health budget) and integrated care partnerships (broader collaborations involving NHS, local government, and other agencies).^13^

The proposed institutional structure differs fundamentally from the internal market, with a focus on integrating different elements of health care such as primary and secondary, hospital and community, physical and mental health, and health and social care. These integrated health services also are to work closely with local authorities. In addition, a new payment system is planned to promote a shift toward population-based funding. Rather than competitive tendering, integrated care boards will be able to award long-term contracts to single organizations.^14^

But policy change is not straightforward. These changes need to reach across an extensive range of agents. Health systems are highly interdependent, built on complex relationships among actors with diverse interests.^15^ Change needs to filter through a “large constellation of forces” where contested relationships between agents, institutions, and processes create a system of “policy inertia”.^16, pp. 83-85^ The wider political climate is also influential.^17^ Moreover, policy implementation takes place in the context of wider structural shifts, such as global capitalism. Neoliberalism, associated with a greater role for market structures and the private sector, increased individualism, and curbing of public spending, has had far-reaching effects on global health systems.^18,19,20^ The rise of neoliberalism has been underpinned by an increase in financialization.^21^ Definitions are wide-ranging, but broadly, financialization refers to the expansion of financial markets, actors, and instruments into more areas of the economy and society, creating new regimes of accumulation and increasing shareholder value.^22^ Financialization in health care services is associated with a growing role for financial actors in social reproduction as they seek new profit opportunities. Research has shown its variegated nature in health systems in Turkey,^23^ France,^24^ lower-income countries,^25^ and England's social care system.^26^ Despite variations, some common themes emerge, with financialization associated with growing inequalities.

This study situates the reforms of the HCB in the context of such wider systemic shifts, drawing on an extensive review of documentation, including sector policy and reports, transcripts of government debates, consultations, and company accounts, beginning with financialization in England's health system.

## Financialization and Shifting Relations

**Figure 1.**
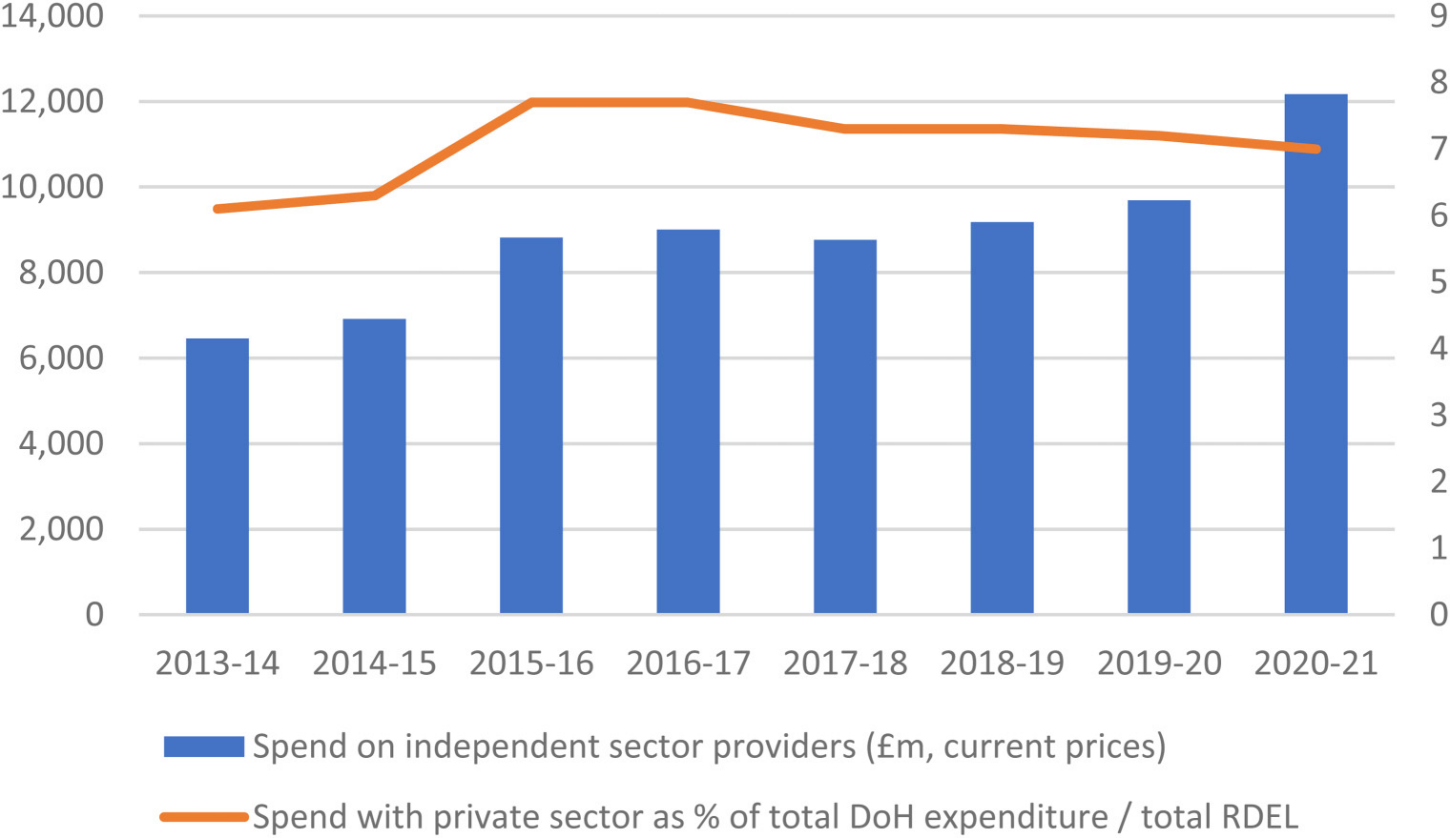
NHS spend on independent-sector providers.

Source: DHSC accounts, various years.

The 2012 HSCA provided for a greater role for the private sector in health services. Figure 1 shows that the amount spent by the Department of Health and Social Care (DHSC) on independent providers has increased from £6.4 million in 2013–2014 to £12.2 million in 2020–2021, with a sharp increase in the last year due to COVID-19. However, the overall share going to the independent sector has remained stable, at around 7% of the DHSC budget. When spending on primary care providers (such as GPs, opticians, pharmacies, and dentists)—which have been private since the establishment of the NHS—is included, spending on independent-sector providers rises to 25%,^27^ but still, the evidence does not indicate a surge in privatization.

But these data fail to capture shifting dynamics within the health system, with diversity across segments of health services and a shift in the nature of investors. The independent sector is a broad term capturing a set of agents with diverse incentives. The DHSC does not provide a breakdown of the independent sector, but the Independent Health Providers Network (www.ihpn.org.uk) includes members that are registered charities, such as Nuffield Health, and those owned by private shareholders, such as Spire Healthcare.

There are some segments of health service delivery where the independent sector is more prevalent, although data on NHS contracts are not easily available. In 2017, almost one-third (29.4%) of NHS-funded knee replacements and 19.7% of hip replacements were carried out by the independent sector, up from almost zero in 2003.^28^ The independent sector is also strongly represented in mental health services, partly because of a sharp decline in the number of mental health beds in England, which fell from 67,100 in 1987 to 18,400 in 2019.^29^ The *Financial Times* reports that private providers manage more than half of mental health beds.^30^ In 2017–2018, 44% (£199 million) of NHS spending on inpatient services for child and adolescent mental health was allocated to the independent sector.^31^ The Care Quality Commission found that 53% of mental health rehabilitation inpatient services were provided by the private sector, and this share rose to 78% of beds categorized as “locked rehabilitation” or “complex care.” The independent sector accommodated patients that stayed for longer than NHS patients, and hence their placements cost more than those of NHS patients.^32^ In community health services, Virgin Care Services Ltd was awarded contracts worth more than £2 billion between 2013 and 2018 to provide multiple mental and community-based health services.^33^

In primary care, GPs are mostly run by clinician-led, individual private partnerships under a standard General Medical Services contract. Since 2004, an Alternative Provider of Medical Services (APMS) contract has been allowed that permits contracts with third-party organizations such as private companies. While the APMS contracts are not widely used, accounting for just 180 of a total of 7001 GP practices in England in 2020,^34^ this is where there have been some radical developments in ownership structures. Some GPs have acquired others to build up chains of practices, two of which (The Practice and AT Medics) were sold in 2021 to a subsidiary of U.S. for-profit health company Centene,^35^ which, according to the campaign group We Own It, now operates 70 GP surgeries in England (1% of the total).^36^ While this takeover is a new development in the nature of private-sector involvement in the NHS, it is rooted in processes set in place during the mid-2000s.

Health service provision has become a financial asset, with ownerships traded as part of global portfolios. For example, private equity owner Cinven bought Spire, one of the largest independent-sector providers of acute, NHS-funded care, from BUPA and then floated the company on the London Stock Exchange in 2014.^37^ Virgin Care was sold in 2021 to private equity investor Twenty20 Capital for an “undisclosed sum”.^38^ In mental health, four companies account for 65% of the private market. Three of these (Cygnet, Elysium Healthcare, and The Priory) are owned by for-profit investors. Cygnet, which provides services for individuals with mental health needs, autism, and learning disabilities, is owned by U.S. health company Universal Health Services Inc. Elysium Healthcare was sold in 2020 by private equity-owned BC Partners to Australian health company Ramsay Healthcare. The largest, The Priory, with 27% of the private mental health market,^39^ was sold by U.S. health company Acadia to a Dutch private equity firm, Waterland, for £1 billion in 2020.^40^

The nature of the private owner is important. Private equity ownership is associated with an aggressive form of value extraction. The finances of companies acquired are usually radically restructured, often creating high debt levels, in part due to leveraged buyouts where the costs of buying the company are allocated to the new corporate structure of the acquisition. Tax liabilities and disclosure requirements are minimized by setting up a parent company in an offshore jurisdiction with low taxes. Companies also benefit from increasing the size of a company as this increases value, so there is a tendency to buy up other practices and businesses to create a chain of provider. Investors prefer secure, low-risk revenue streams.^41^

The Priory demonstrates some of these core financialization practices. Since it was founded with a single hospital in Roehampton in 1980, The Priory has been sold and resold several times to and from private equity and financial investors, expanding and being loaded with more debt, as demonstrated in the accounts of the holding company, Priory Group No. 1 Ltd Accounts (various years). Following the sale to Acadia in 2016, company debts rose immediately to £1.1 billion (from £420 million in 2015). The debts were owed to a holding company registered in Jersey attracting annual interest at 7.4%. In 2020, £84 million—equivalent to nearly 10% of revenue (90% of which is from the NHS and local authorities)—was paid in interest on intercompany debt to the holding company. While no dividends were paid in the five years of Acadia's ownership, total interest paid on intercompany loans over this period came to £428 million. Interest payments contributed to the company's accumulated losses, totaling more than £1 billion in 2020.

Some financial investors have capitalized on the security of rental payments in NHS-funded health services, separating these from the operation of the facility. In order to sell the debt-laden company, some of The Priory's property was sold separately to a U.S.-based real estate fund, the Medical Properties Trust, and leased back to The Priory. Under this arrangement, The Priory is reported to be committed to rental costs of about £50 million a year and a minimum annual rent increase of 2%.^42^ Sale and leaseback arrangements also feature in primary care, with GP premises bought from practice partners and leased back to them under a management contract. Assura Plc owns 609 GP properties, about 6% of the GP premises in the United Kingdom.^43^ Approximately 84% of rental income is from GPs or NHS bodies. The company paid dividends of £74 million in 2021 (£66 million in 2020). The majority shareholder is Blackrock, with 11% of shares. Leases are typically more than 21 years in length and reimbursed in full by the NHS.^44^

Financialization creates complex corporate structures that compromise transparency in accounting for NHS funds. Complex inter-group transfers, often via tax havens, make it impossible for an outsider to trace the financial flows. Virgin Care was headquartered in the British Virgin Islands, Elysium is owned by a company registered in Luxembourg, and The Priory was owned via a Jersey-based holding company. In one complex corporate transaction, England's largest^45^ independent provider of NHS services, Care UK, was sold to another, the Practice Plus Group, both of which are owned by funds managed by private equity firm Bridgepoint, using loan finance and equity provided by Bridgepoint. The parent of Practice Plus is Bridgepoint Europe Portfolio IV LP.^46^ This limited partnership is one of numerous funds managed by Bridgepoint. Filings for this partnership at Companies House^1^ show that investors are themselves private equity funds, mostly registered in tax havens such as Delaware and the Cayman Islands, although the Pensionskasse Stadt Zurich City of Zurich pension fund is also an investor. Thus, payments from the NHS flow through complex layers of financial agents to opaque, offshore destinations.

These examples show how tax-funded payments by NHS England for essential health services are sustaining an extensive, global financial architecture, including real estate, and reaching through a complex corporate web into global circuits of finance capital. The process leads to a shift and expansion in the agency relations that underpin publicly financed health care services. Private finance is drawn to the secure revenue streams offered by NHS funding, which allows it to push debt levels further to increase returns to shareholders and to buy up property with the promise of rent to be paid from NHS funds. These financial structures mean that NHS funds are diverted to offshore tax havens via interest payments that lower tax liabilities, creating financial vulnerabilities..^26^

**Figure 2.**
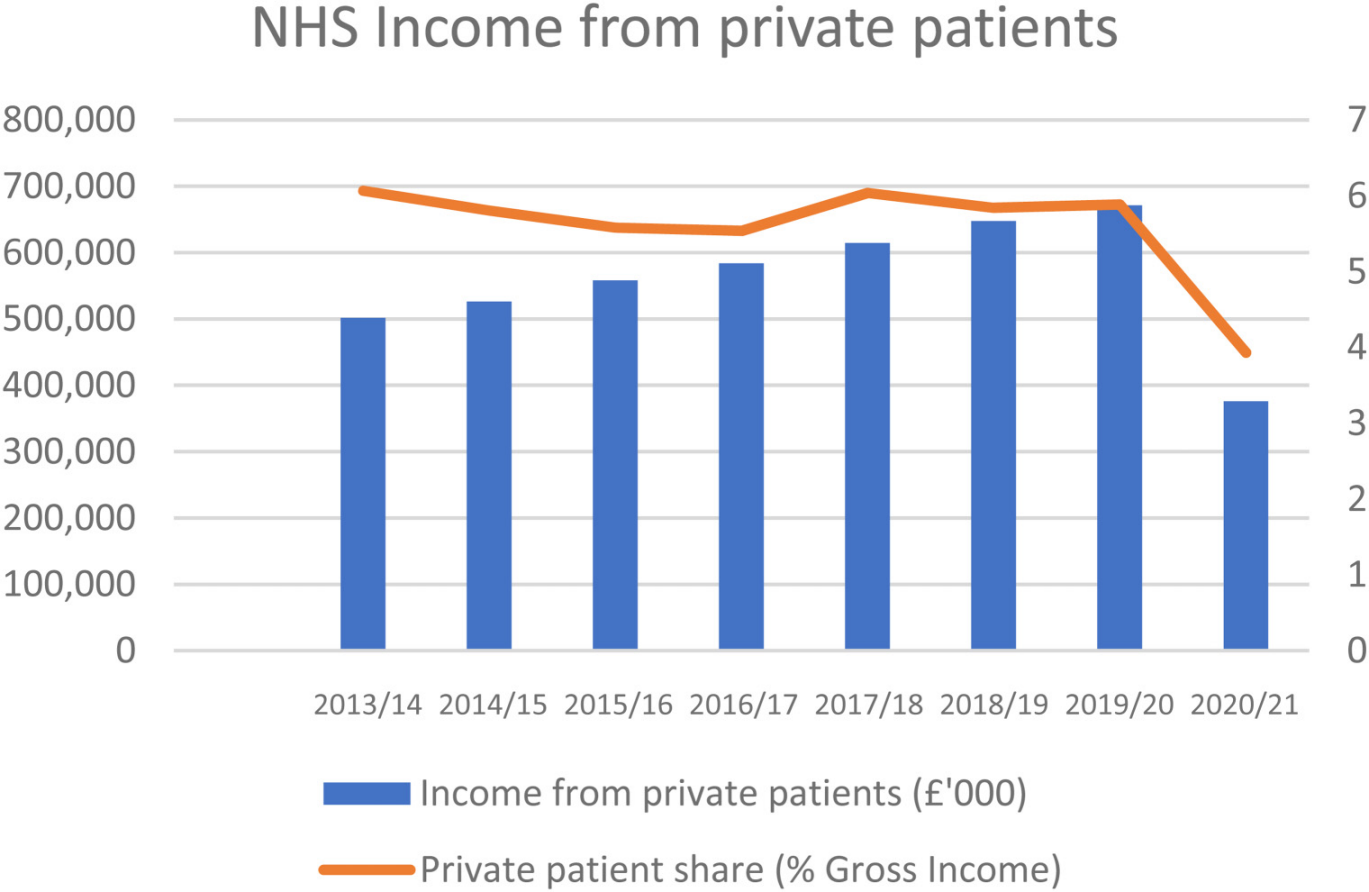
NHS income from private patients.

Source: DHSC accounts, various years.

The intersection between the state and market in health services has been further complicated by a growing scope for the NHS to earn revenue from private, fee-paying patients. The HSCA 2012 provided for a lifting of the cap that NHS providers could earn from private patients, from 2% to 49% of income share. Numerous hospitals established private-patient units, and the income from private patients was expected to become a significant source of NHS hospital trust income.^47^ Figure 2 shows that the NHS revenue from private patients was increasing before the pandemic, but the overall share of NHS revenue that is from private patients has changed little, accounting for less than 6% of NHS income.

There is large, regional variation, with the share of income from private patients much higher for London hospitals than those in the rest of England. But the legal and institutional framework has been established across the country, and NHS hospitals are marketing private-patient units on the grounds that it helps the NHS.^48^ Moreover, media sources suggest that recent guidance encourages NHS providers to develop private-patient services in order to generate revenue.^49^ While private income for the NHS has not greatly increased, new partnerships are emerging between state and private providers. For example, the Christie Private Care Partnership is a partnership between the Manchester-based Christie NHS Foundation Trust and U.S. health company HCA International Ltd The partnership was established in 2010 for the provision of private oncology activity and contributed £4.1 million to NHS Foundation Trust accounts in 2020.^50^ This arrangement brings private health providers closer to the public health system. The NHS is listed by the market analysts LaingBuisson as a provider in the private health market, thereby complicating its character as a public-sector institution.

## The Wider Context: Austerity and Covid-19

Demand for health services tends to increase due to a growing and aging population, rising costs, expectations, admissions, and prevalence of long-term conditions. Finance has not grown consistently (Table 1).^51^

**Table 1.** Growth in Government Spending on Health Care Services .^51^

Period	Financial years	Real health spending growth rate (%)	Real health spending per capita growth rate (%)
Pre-1979 governments	1949/50–1978/79	3.5	3.1
Thatcher and Major governments	1978/79–1996/97	3.3	3.1
Blair and Brown governments	1996/97–2009/10	6.0	5.5
Coalition government	2009/10–2014/15	1.1	0.3
Conservative governments	2014/15–2019/20	2.2	1.5

Source: Warner M and Zaranko B. *Pressures on the NHS*, Institute for Fiscal Studies. 2021.

When the Conservative–Liberal Democrat Coalition government entered power in 2010, waiting times were the lowest they had ever been. International surveys put the NHS high in the league tables on many measures, public satisfaction was at its highest level ever,^52^ and the United Kingdom compared favorably to other health systems, such as that of the United States.^53^ But funding growth then slowed dramatically. NHS providers shifted from a position of surplus to a substantial deficit.

The financial stress led to immense pressure on the health system, with providers expected to do more with less. Unrealistic targets were consistently missed, leading to financial penalties that exacerbated financial pressures. With more than 60% of total expenditure accounted for by staff costs, the workforce was severely affected (and compounded by Brexit). NHS trusts were advised to review staffing so that only essential vacancies were filled. A 1% pay cap imposed on health-sector workers in 2010 led to a substantial fall in real wages for many. Increased use of high-cost agency staff added to financial pressures.^54^

Meanwhile, demand was increasing. Between 2016 and 2019, the NHS faced average annual growth in elective referrals of more than 2%. Between 2010 and 2019, there was an annual average growth in emergency admissions of more than 3% and a growth of more than 10% in urgent cancer referrals from GPs. Within this context, the NHS managed to treat more patients each year, particularly for cancer. But the level of increase was not sufficient to keep pace with demand.^55^ The waiting list for elective care grew from 2.9 million to 4.4 million between 2015 and 2019.^56^ In March 2017, some of the targets for waiting times were “relaxed”.^57^

Compared with other Organisation for Economic Co-operation and Development countries, the United Kingdom entered the COVID-19 pandemic with relatively weak capacity, as demonstrated by low numbers of hospital beds, nurses, and doctors per 1000 population and higher levels of bed occupancy than comparable health systems.^58^ Two years later, the health sector is in an even weaker state. In December 2021, the waiting list for elective care reached 6.07 million, the highest level since records began, and numbers are expected to increase.^55, 59^ The pandemic is anticipated to lead to a sharp increase in demand for mental health services.^60^ Staff pressures have been exacerbated, and 36% of nurses were reported to be thinking of leaving the profession in June 2020.^51^ In 2021, there were 93,000 vacancies for NHS, and workforce shortages are considered to be the “key limiting factor” in addressing the backlog in NHS elective care.^61^

Funding levels were due to be increased in the 2018 five-year funding settlement to provide a real-terms increase of 3.4% a year, but this was blown away by COVID.^51^ More funding has been pledged. In September 2021, the government announced that an additional £36 billion would be invested in health and social care across the United Kingdom over the three years from 2022–2023 to 2024–2025.^55^ While more funding is promised, effects are lagged, and evidence presented to the House of Commons Select Committee suggests it will be two to three years before a material increase in NHS capacity occurs.^59^

But while the pandemic had a negative impact on the NHS, it provided a much needed boost to private health care services in England. The private acute market for medical care declined (in terms of revenue) by 2.1% in 2017 and 1.1% in 2018 due to “the stalling of key funding streams” as NHS spending on the independent sector fell in 2017 and 2018. Meanwhile, medical insurance payouts were stagnant. An increase in self-pay patients was not enough to compensate. Some hospitals were closed.^62^ The number of acute beds in the independent sector has been falling gradually from a peak in the mid-1990s.^63^

However, starting in March 2020, NHS England made a series of contracts with most of the private hospitals in the country that gave it access to all their facilities, staff, and equipment in order to deal with the COVID response. While the contracts were modified over the course of the pandemic, in the 2020–2021 financial year, the NHS paid private, independent providers approximately £2.1 billion under these special contracts.^55^ The national Increasing Capacity Framework is expected to spend up to £10 billion between 2021–2022 and 2024–2025 for a framework agreement with more than 80 independent providers.^55^ An additional three-month “surge” deal in January 2022 meant a list of independent organizations would put their staff on standby to support the NHS in the event that Omicron would lead to unsustainable pressures. In February 2022, the NHS Elective Care Recovery Plan set out a clear role for the private sector.^64^ The pandemic also led to new partnerships with private providers. For example, London NHS hospital trusts bought £36 million of cardiology and cancer care services from HCA International in 2021. Prior to the pandemic, HCA carried out virtually no work for the NHS.^65^

Yet for much of the COVID period, while private hospitals were paid their full operating costs minus the cost of any capacity the NHS agreed to release for the treatment of private patients, most of this was not used. In practice, there is little capacity that private providers can bring in the context of the pandemic. Most private hospitals are small, without high dependency or intensive care facilities. During the year to June 2021, a total of 518 patients were transferred to NHS facilities for urgent care following complications during treatment in the independent sector, a rate of about one per 1000 patients treated.^66^ Private hospitals use the same senior doctors, surgeons, and anesthetists as those that work in the NHS so value of additional capacity provided can be overestimated.^67, 68^ While the impact on NHS capacity was negligible, these deals were vital for some private providers.

**Figure 3.**
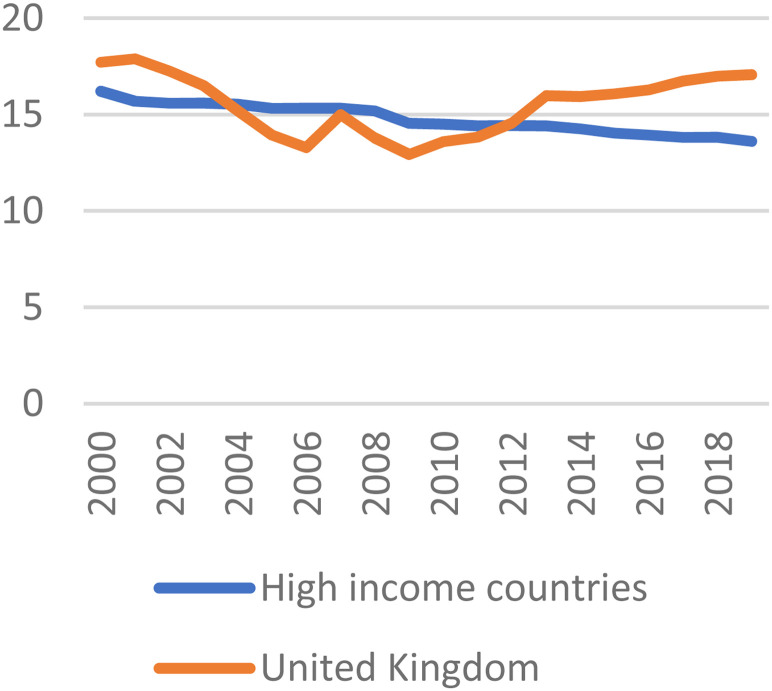
Out-of-pocket expenditure (% current health expenditure)^78^

source: World Bank Data.

The private sector has also seen a major boost from people paying for health services out-of-pocket to avoid lengthening waiting lists. Figure 3 shows that the share of health expenditure that is out-of-pocket had declined since 2000, but this trend was reversed in 2010. In the longer term, out-of-pocket spending has risen faster in the United Kingdom since the 1970s than in any other G7 nation.^69^

Out-of-pocket expenditure as a share of health expenditure and self-funded health services have surged in the wake of the pandemic. Comparing data for July to September 2021 with the same period in 2019, self-funded hip replacements increased 165%, knee replacements 122%, and cataract operations 64%.^70^ Spire saw a decline in revenue from medical insurance in 2020 compensated for by a boost in NHS revenue. This was followed by “unprecedented demand” from self-paying patients, many of whom have not used private health care before, resulting in a 46.7% increase in revenue.^71^ Years of state-orchestrated decline in public capacity, combined with generous contracts under the pandemic, have strengthened private health care as a profitable business.

## Implications for the Health and Care Bill

The major restructuring of the NHS needs to be understood in terms of these ongoing dynamics. Rather than a question of more or less privatization, state and market are closely connected, but with variegation within each of these categories. The nature and role of private-sector involvement in NHS-funded services has evolved across different segments of the service. For example, digital health technologies offer scope for increased private-sector involvement, including, with Amazon.^72^ Canadian private equity firm Impala, taking advantage of workforce shortages, owns several staffing agencies (Thornbury Nursing Services, Pulse, Bank Partners, CHS Healthcare Holdings) via Acacium Group Holdings Limited registered in Jersey.^73^

Increasing financialization has led to an increase in the range of agents that are seeking to profit from the NHS and are attached to different elements of NHS-funded services, often via global financial centers. Ownerships have become consolidated, such as with Centene, which now operates across a substantial share of primary and secondary care. The private sector is deeply embedded in some elements of health services, particularly where a lack of NHS capacity has created a reliance on the private sector, as in mental health. Some companies, such as Spire, Circle, Ramsay, and Practice Plus, carry out a large number of routine, elective NHS interventions and primary health care. For Ramsay, 78% of admissions were from the NHS in 2019.^74^ For some mental health providers, the share of income from the NHS is even higher. Almost all revenue for Cygnet Healthcare is from the NHS and local authorities.^75^

There is, then, an increasing private-sector voice, and independent-sector providers do not want to be excluded from the new NHS institutional structures. The IHPN, a membership-based advocacy group for independent health providers, states that it is continuing work related to influencing emerging NHS integrated care systems and ensuring independent providers are fully factored into their plans.^76^ While the amendment to the HCB indicates that the private sector is at present unlikely to feature in the boards of integrated care systems, they are expected to continue to play a significant role in health provision, both within the NHS and for the growing population paying for private treatment.

The pandemic led to a surge in public health spending, more so in England than in other countries due to the underlying weaknesses in the system.^58^ However, the long-term health spending trajectory is similar to that of comparable countries.^77^ But such levels of public spending do not sit comfortably with the current political regime. Secretary of State Sajid Javid, in a March 2022 speech, stated his position as a “small-state conservative,” depicting the health service as unaffordable, with current monthly spending equivalent to the 1948 annual budget and a current health budget that is “now bigger than the GDP of Greece”.^78^ Javid paves the way for lower expectations of the NHS, anticipating increased demands on households to fill the gaps in public services, stating “there's no small state without strong families.”

The political landscape seems, then, to be strongly supportive of the private sector, and there are close connections, such as with the Centene UK Chief Executive in 2019 appointed Downing St Chief Operating Officer in February 2022.^79^ Political connections also played a role in the award of contracts during the pandemic.^80^ The indications are that the private sector will continue to play a significant role in health services, which raises a number of concerns for the future of the NHS.

First, the private sector has a preference for the easiest-to-treat patients and the most profitable activities, leaving the rest to the state. Spire Healthcare indicated that the Increasing Capacity Framework puts it under pressure to treat patients who have been on NHS waiting lists the longest, but these are likely to be more challenging with higher levels of acuity. They suggest that they are better placed to provide “high volumes of low-complexity activity”.^81^ One chain of GPs, Integral Medical Holdings, which generates most of its revenue from providing services to NHS patients, operating a network of 11 GP practices, and delivering after-hours services, is planning to exit unprofitable GP practices.^82^ This tendency to cherry-pick the most lucrative and least demanding activities risks creating a fragmented structure with more rather than less demands on the NHS. Moreover, company decisions to close services on commercial grounds creates vulnerabilities for the NHS. For example, the number of mental health care beds for teenagers declined by about 20% in the pandemic due to staff shortages and increased acuity of cases.^30^

Second, the private sector operates according to commercial structures that do not fit with the social objectives of the NHS and can increase costs—for example, seeking predictable revenue streams. Spire has suggested that contracts with the NHS of three to five years’ duration with guarantees of minimum levels of activity will provide the confidence to invest.^82^ Contracting private capacity to deal with a potential surge in cases due to the Omicron COVID variant required the NHS to provide a minimum income guarantee, “creating a material risk that the NHS pays for activity that is not performed” and leaving the health service “financially exposed”.^83^ The proposed new NHS funding system is oriented around public health management with a focus on prevention of disease.^84^ But this does not sit comfortably with the commercial priorities of the private sector, which has a preference for the old “tariff” system, with funding attached to a specific procedure.^76^

Third, there are many ways in which the interests of the private-sector and public health systems are incompatible. The private sector seeks to increase revenues. This feeds into numerous transactions across different layers of private-sector involvement. For example, Assura Plc generates revenue from NHS-funded rental payments for GP premises and is targeting a growth in rental income from rent reviews that is greater than inflation.^44^ On a more systemic level, what is bad for the NHS is often good for the private sector and vice versa. Growing waiting lists benefit the private sector, where they lead to more self-pay patients and more outsourcing to private providers. Measures introduced in 2017 to improve communication between GPs and consultants led to a reduction in NHS costs due to a sharp fall in referrals for elective health care for all health providers.^85^ However, this was experienced as a more challenging market for the private sector and cited by South Africa investor Netcare as a reason for leaving the UK health sector.^86^ In mental health, private providers gain from a culture of detentions for patients boosted by a “fear factor” ^87^ that is not necessarily in the interests of patients.

Fourth, the identity of the contractor can change as ownerships are traded. Virgin Care was awarded a three-year extension to a contract to provide a range of community health services in Bath and South West England in November 2021,^88^ and the next month, Virgin was sold toTwenty20, a private equity firm with a different corporate ethos. Virgin Care, despite its private ownership, did not make profits from its investments in NHS contracts, stating its commitment to reinvesting back into the company and frontline health services. In contrast, the new owners state that they “look for significant returns in 2–5 years”.^89^

Finally, the private health sector's involvement in health care services risks increasing inequalities. A long-term decline in service provision, exacerbated by the pandemic, has led to a surge in self payment as an opt-out for those who have the means. This trend “threatens to define the NHS's “new normal,”” leading to a two-tier system similar to that which exists for dentistry.^90^ ^p. 26^ In addition, private equity investment generates generous returns for investors. Financialization means that public funds flow to finance capital through payments of rent, interest, and dividends and often circumvent tax rules. A government commitment to raise funding for mental health, for example, is expected to be a boost for mental health providers, with the market also buoyed by lack of NHS capacity.^87^ Some private investors in NHS-funded health services are among the world's richest,^91^ for whom England's health sector is a small cog in a multisector, global, revenue-generating investment portfolio.

## Conclusion

The ongoing reforms to the NHS present a major shift in policy and ethos with an integrated approach to managing population health. But the outcomes will be shaped by the context. The legacy of the neoliberal history has created a complex and contradictory set of pressures on the NHS that risk undermining the reform goals. The nature of the private sector has shifted with the expansion of for-profit investors extracting revenue via financial engineering. U.S. health companies now own a share of primary care provision. Financialization is deeply embedded in some segments of health services as long-term structures intersect with global processes. The NHS itself is also conflicted, operating in part as a provider of private services. There is a potential for cash-strapped public health providers to boost budgets with fee-paying patients. Public provision is failing. Waiting lists for health services are at record highs. The government is attributing this to COVID-19. For example, the plan to tackle waiting lists is titled the “Delivery Plan for Tackling the COVID-19 Backlog of Elective Care”,^64^ but most of the backlog stems from before the pandemic. The private sector is picking up the pieces. Moreover, while the policy is shifting toward an integrated public health system, some important aspects of the neoliberal framework remain, such as patient choice.^11^ Contrary to the narrative of cooperation, the policy of patient choice fosters the idea of the patient as a consumer and retains an element of competition among providers, although the benefits for citizen welfare are unclear.^48^

The fortunes of the private health “market” are closely tied to the activities of the state. There are parallels between the current situation and the long waiting lists in the early 2000s, which the Labour Government managed to reduce substantially. But the context has changed with a well-established private sector, an increasingly normalized self-pay culture, and a government that is showing no signs of raising spending to the 6% growth levels of the Blair/Brown years. Rather, the government has been strongly supportive of the private sector in its response to COVID-19, and the health secretary has stated his commitment to a small state.

The HCB does not set out a clear policy for greater private-sector involvement, but equally, it does not state a commitment to the NHS as the preferred provider of services. Given the extensive role of the private sector in some areas, integrated care is bound to include the private sector. In some cases, this has already begun—for example, the Bath and North East Somerset, Swindon, and Wiltshire integrated care partnership includes Virgin Care alongside several NHS Trusts and local councils.^92^ While this is some distance from the commissioning integrated care board, this is still a position of influence.

The NHS has strong cultural associations, featuring in the opening ceremony of the 2012 London Olympics, but cultures are changing and anti-NHS rhetoric is growing, such as with hostility to the 1.25% Health and Social Care Levy added to national insurance contributions in April 2022.^93^ NHS staff are reporting an increase in abusive behavior from patients, with a reduction in tolerance from patients for delays in treatments.^94^ The narrative is promoted that opting to pay for services out-of-pocket will reduce pressure on the NHS, when in practice it helps to normalize access to health services based on income.

Campaign groups have organized around opposition to the privatization of the NHS. But resistance is muted, in part because of the density of the underlying dynamics. Privatization in terms of the expansion of the private provision of NHS services has been limited, which means claims of privatization are easily dismissed. But rather than traditional privatization, there is more subtle and pernicious erosion of the public health system across different dimensions. Agency relations have shifted. The NHS now operates in close partnerships with private companies and is now a private health provider itself. A powerful private sector has evolved, partly providing NHS-funded services but now capitalizing on shortages in the public system. In the absence of a substantial boost to NHS funding and a commitment to public provision, neither of which seem likely in the current political climate, the reforms of the HCB will be shaped by this pre-existing context in ways that risk severely undermining the policy ambitions of an integrated care system.

## Notes

1. In particular, the LP6 filings on changes in the Limited Partnership
